# Pigmented Corn Varieties as Functional Ingredients for Gluten-Free Products

**DOI:** 10.3390/foods10081770

**Published:** 2021-07-30

**Authors:** Francesca Colombo, Chiara Di Lorenzo, Katia Petroni, Marco Silano, Roberto Pilu, Ermelinda Falletta, Simone Biella, Patrizia Restani

**Affiliations:** 1 Department of Pharmacological and Biomolecular Sciences, Università degli Studi di Milano, 20133 Milan, Italy; francesca.colombo1@unimi.it (F.C.); chiara.dilorenzo@unimi.it (C.D.L.); simone.biella@unimi.it (S.B.); 2 Department of Biosciences, Università degli Studi di Milano, 20133 Milan, Italy; katia.petroni@unimi.it; 3 Unit of Human Nutrition and Health, Department of Food Safety, Nutrition and Veterinary Public Health, Istituto Superiore di Sanità, 00161 Roma, Italy; marco.silano@iss.it; 4 Department of Agricultural and Environmental Sciences—Production, Landscape, Agroenergy, Università degli Studi di Milano, 20133 Milan, Italy; salvatore.pilu@unimi.it; 5 Department of Chemistry, Università degli Studi di Milano, 20133 Milan, Italy; ermelinda.falletta@unimi.it; 6 CRC “Innovation for Well-Being and Environment”, Università degli Studi di Milano, 20122 Milan, Italy

**Keywords:** celiac disease, oxidative stress, polyphenols, pigmented corn, *Zea mays* L.

## Abstract

Oxidative stress, one among the several factors responsible for the gluten toxicity in celiac disease, together with inflammation and duodenal mucosal injury, are only partially reduced by the gluten-free diet. Thanks to their phenolic profile, the pigmented varieties of corn could be an interesting source of dietary antioxidants for the formulation of new gluten-free ingredients. The aim of this research was: (1) to characterize the phenolic profile and the associated antioxidant properties of corn samples with different pigmentation, using spectrophotometric and chromatographic techniques and (2) to assess the stability of anthocyanins during the gastro-intestinal digestion. The pigmented varieties showed a significantly higher content of polyphenols compared to the common yellow varieties and, as a consequence, a higher antioxidant activity. Although corn is among the cereals most frequently used in gluten-free products, it can produce an inflammatory response in some celiac patients. Therefore, after the chemical characterization, the safety of the pigmented varieties for celiac patients was confirmed using different in vitro models (cell agglutination test and the measure of transepithelial electrical resistance). Although in vivo studies are necessary, the data collected in this study underline that the pigmented corn could have a role in reducing the oxidative stress at the intestinal level in celiac subjects.

## 1. Introduction

In the last years, an increased prevalence of gluten-related disorders (GRDs) has been observed. Celiac disease (CD) is a wide health problem with a global prevalence of 1.4%, based on serologic testing, and of 0.7%, based on a biopsy investigation [1]. A gluten-free diet (GFD) is the only currently available treatment for CD, but a long-term GFD could cause some nutritional deficiencies and lead to a caloric-nutrient imbalance [2,3]. In fact, gluten-free deriving products are usually low in fiber, certain minerals, vitamins [4,5,6] and rich in lipids, sugars, and salt [7].

In this contest, an improvement in gluten-free products, in term of both nutritional and sensory aspects, is welcome.

CD is an immune-mediate disorder, triggered in genetically predisposed individuals by prolamins (wheat gliadin, rye secalin, and barley hordein). Some gliadin sequences are responsible for cytotoxic or immunomodulatory activity, while other regions are responsible for the oxidative stress at intestinal level [8]. The gliadin peptides enter into the cells causing the production of pro-inflammatory cytokines and enzymes (such as inducible nitric oxide synthase—iNOS), which increase the production of nitric oxide (NO). NO is a metabolite promoting oxidative stress and a greater mucosal permeability mediated by the alteration of gut barrier function. Some authors suggested that patients on a strict GFD showed a pattern of ongoing disease, in terms of inflammation and persistent villus atrophy [9,10]. Moreover, the oxidative imbalance, identified as one of the pathogenetic mechanisms of CD [11,12,13], seems to be only partially reverted by the gluten-free diet [14,15].

In addition, reactive oxygen species are produced into the celiac enterocytes exposed to gliadin cytotoxic peptides, through a mechanism that involves the activation of cellular transglutaminase-2 and the subsequent ubiquitin derangement [16]. So, in the CD inflammatory cascade, a key early role is played by the oxidative stress. 

Fruits, vegetables, and cereals contain polyphenols with well-known antioxidant and anti-inflammatory properties, involving (1) the reduction of the activity of different enzymes, such as phospholipase A2, cyclooxygenase, and lipoxygenase; and (2) the inhibition of iNOS activity and expression, as observed in different in vitro models [8]. Some epidemiological and population-based studies have suggested that the consumption of foods rich in polyphenols may contribute to the reduction in risk factors for chronic diseases. Although more in vivo studies are necessary, polyphenols could be a safe alternative and a natural strategy to modulate the pathological pathways associated with CD [17]. 

Corn and rice are among the ingredients most frequently used for the formulation of gluten-free products. The pigmented varieties of corn, originating in South America, are still not widespread in Europe. Being rich in anthocyanins, which are known for the associated antioxidant and antiradical activities [18,19], they could be used as healthy ingredients for the formulation of novel gluten-free products. In most CD patients, symptoms disappear after a strict GFD; however, in some celiac subjects, the symptoms persisted. This could be due to refractory CD or to the presence of gluten contaminations. However, some authors suggested that corn could determine an inflammatory response in a very limited subgroup of celiac subjects [20]. 

All these findings highlight the importance to evaluate “new” corn varieties for their capability in activating the epithelial cells in CD, before their inclusion as an ingredient in gluten-free products.

Considering the possible role of polyphenols in modulating the critical regulatory pathways associated with CD, in this study new pigmented corn varieties were compared with non-pigmented varieties, to propose their use as ingredients of polyphenols’ rich food, for which the safety for celiac patients was confirmed. The safety for celiac subjects was tested using different cellular tests (cell agglutination test and the measure of transepithelial electrical resistance). Subsequently, the functional properties of pigmented corn were assessed, by performing their chemical characterization and studying the antioxidant activity. The stability of active molecules through the digestive tract is of primary importance to support their health properties. Although the stability of anthocyanins during human digestion has been evaluated for different foods, the studies on pigmented cereals were mainly focused on extracts or pure anthocyanin compounds [21]. In this study, an in vitro model was used to mimic human digestion and evaluate the stability of anthocyanins in corn flour during transit in the gastro-intestinal tract.

## 2. Materials and Methods

### 2.1. Chemicals and Reagents 

Folin-Ciocalteau reagent, 1,1-diphenyl-2-picryl-hydrazyl free radical (DPPH), trolox, potassium persulfate, 2-2′-Azino-bis-(3-ethylbenzthiazoline-6-sulfonic acid) (ABTS), salts, enzymes, and standards of phenolic acids were purchased from Sigma Aldrich (Steinheim, Germany); ethanol, methanol, acetone, toluene, and formic acid were from VWR International (Fontenay-sous-Bois, France); standards of anthocyanins were from Extrasynthese (Genay, France).

### 2.2. Samples

Seven varieties of *Zea mays* L. with different genetic characteristics were included in this study: one synthetic purple corn variety carrying B1 and Pl1 alleles, here called as Reduno (used for animal feeding), three yellow varieties (suitable for human consumption), and three pigmented varieties obtained through recurrent backcrosses between the above-reported purple and yellow varieties. The crossbreeding process was performed to obtain pigmented varieties suitable for the human diet and for the cultivation in temperate climates [19].

**Figure 1:**
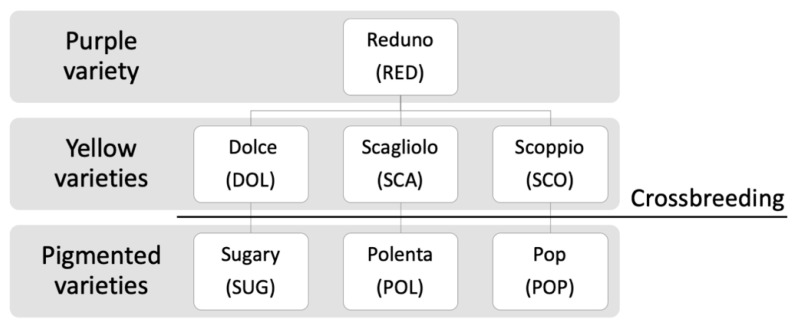
Corn varieties included in the study and relative abbreviations.

Corn kernels were finely ground by a gluten-free dedicated coffee mill, until able to obtain a homogeneous flour, and stored at 4 °C. The samples and identification codes used in this study are reported in Figure 1.

### 2.3. Cellular Tests as Models for the Evaluation of the Safety of Pigmented Corn Varieties in Celiac Patients

#### 2.3.1. In Vitro Peptic-Tryptic Digestion 

In order to confirm the safety of pigmented corn varieties for CD patients, two pigmented corn were selected for this preliminary test: Reduno and Polenta. Proteolysis of corn, wheat (positive control), and rice (negative control) flours was performed using purified pepsin and trypsin [22]. The digestion was performed adding 25 mL of 0.1 N HCl (containing 5 mg of pepsin from porcine gastric mucosa) to 2.5 g of each sample. Samples were incubated at 37 °C for 2 h. At the end of the gastric digestion, the pH was adjusted to 8.0 with 0.2 N NaOH, and 5 mg of trypsin from bovine pancreas were added. The samples were incubated for 2 h at 37 °C. At the end of the incubation time, the pH was adjusted to 7.0 with 0.1 N HCl, and samples were freeze-dried (freeze-dryer Edwards Modulyo, UK). 

#### 2.3.2. Cell Lines 

Human leukemic K562(S) and human colon adenocarcinoma T84 cell lines were from American Type Culture Collection (LGC Standard, Milan, Italy) and cultured as previously described [23]. 

#### 2.3.3. K562(S) Cell Agglutination Test 

The K562(S) cell agglutination test was performed according to the method described by Silano and co-workers [22,23]. Briefly, K562(S) cells were harvested by centrifugation and washed twice with Ca^2+^ and Mg^2+^ phosphate-buffer saline (PBS, GIBCO, Carlsbad, CA, USA) and resuspended at a concentration of 10^8^ cells/mL in the same buffer. Aliquots of 25 μL of cell suspension were added to each well of a 96-well microtiter plate containing the cereal digestion products at the concentration of 7 mg/mL (final total volume 100 μL). The cell suspension was incubated at room temperature (RT) for 30 min. Then, an aliquot of cell suspension was drawn out from the well and put on a microscope slide. The number of agglutinated cells every 100 cells was counted at the optic microscope, magnification 100×.

#### 2.3.4. Measurement of Transepithelial Electrical Resistance (TEER) across T84 Cell Monolayer 

The cell monolayer was obtained by seeding the T84 cells on polycarbonate inserts (0.45 mm pore diameter, 0.9 cm^2^ area, pore density 1 × 10^8^/cm^2^; BD Falcon, Franklin Lakes, NJ, USA), and it was left to grow to full confluence for at least 19 days. The formation of the cell monolayer was verified by measuring a transepithelial electrical resistance (TEER) value of at least 800 Ω ms/mm^2^, using a Millicell ERS device (Millipore, Bedford, MA, USA). The T84 cells were incubate for 3 h with the digestion products of different cereals (1 mg/mL at 37 °C). The variation of the monolayer permeability, inducted by the digested products, was expressed as a difference in the TEER value measured after 3-h incubation with the digestion product, and the TEER value assessed just before its addition to the cell culture (ΔΩ ms/mm^2^).

### 2.4. Determination of Soluble Phenolic Content

Corn contains phenolic compounds in two forms: a soluble form and bound-to-cell-wall components. This study was focused on the soluble fraction, which is abundant in pigmented varieties. 

#### 2.4.1. Extraction of Soluble Phenolic Compounds

Soluble phenolic compounds were extracted according to Lopez-Martinez and co-workers [24] with some modifications: 0.5 g of ground whole grain were extracted with 10 mL of an ethanol:water 80:20 (*v*/*v*) mixture and maintained under stirring for 2 h in the dark at room temperature. Samples were centrifuged at 2000× *g* for 15 min at 4 °C (Avanti J-25, Beckman Coulter, CA, USA). The supernatant was collected, evaporated using a Rotavapor, freeze-dried, and finally resuspended in 10 mL of water. The extracts were passed on 0.45 μm filters (VWR International, Fontenay-sous-Boys, France) and stored until use at −20 °C. Each extraction was performed in triplicate.

#### 2.4.2. Folin–Ciocalteau’s Assay

The soluble polyphenol content was quantified using Folin–Ciocalteau’s method, as previously described by Singleton and Rossi [25].

Aliquots of 300 μL of samples suitably diluted (or water for blank) were mixed with: 1.5 mL of 0.2 N Folin–Ciocalteau’s reagent and 1.2 mL of 7.5% sodium carbonate. After 30 min in the dark, the absorbance was measured at 765 nm in a UV–visible spectrophotometer (Varian Cary 50 SCAN, Palo Alto, CA, USA). The soluble polyphenols were determined using a calibration curve of gallic acid (from 5 to 50 μg/mL), and the results were expressed as equivalents of gallic acid (GAE) in mg/g. Each analysis was performed in triplicate.

### 2.5. Evaluation of Antioxidant Capacity

#### 2.5.1. DPPH Method

The antioxidant activity (AOA) was determined as a measure of radical scavenging activity using the DPPH spectrophotometric assay [26]. Samples (0.5 mL), prepared as described in Section 2.4.1 and properly diluted, were mixed with 1 mL of 0.005% DPPH in methanol. The absorbance was measured after 30 min at 517 nm, maintaining samples in the dark. The concentration of antioxidants was calculated using a calibration curve of gallic acid (from 1.0 to 5.0 μg/mL) and expressed as equivalents of gallic acid (GAE) in mg/g.

#### 2.5.2. TEAC Assay

The Trolox Equivalent Antioxidant Capacity (TEAC) assay was performed as described by Re and co-workers [27] with some modifications. Solutions of 2.45 mM potassium persulfate and 7 mM ABTS were mixed (1:1 *v*/*v*) and maintained for 12–16 h in the dark at RT, in order to obtain the ABTS radical cation solution.

Before use, the ABTS^+^• solution was diluted with ethanol to reach an absorbance of 0.7 ± 0.02 at 734 nm. Samples (150 μL), prepared as described in Section 2.4.1 and properly diluted, were mixed with ABTS^+^• solution (1.5 mL), and the absorbance was measured at 734 nm after 6 min. The results, expressed as mg/g of Trolox equivalents (mg TE/g), were calculated using a calibration curve in the range of 10–30 μg/mL.

#### 2.5.3. High Performance Thin Layer Chromatography (HPTLC)

The HPTLC technique was used to obtain the fingerprint of samples [28], assessing in parallel the antioxidant capacity associated with any specific compound.

The method for the semi-quantitative evaluation of antioxidant capacity is here described [29].

- Sample preparation
  Soluble phenolic compounds were extracted as previously described (Section 2.4.1), but dried supernatant was suspended in 0.5 mL of methanol.

- Chromatographic conditions
  Aliquots of 5 μL of standard solutions of phenolic acids (coumaric acid and ferulic acid) at the concentration of 200 μg/mL were applied on silica-gel plates 254F (10 × 20 cm, Merck, Darmstadt, Germany), in parallel to corn samples (10 μL) using a semi-auto/sample applicator (Linomat 4, CAMAG, Muttenz, Switzerland). The chromatographic run was conducted using 10 mL of acetone:toluene:formic acid 4.5:4.5:1 (*v*/*v*/*v*).

At the end of the chromatographic run, the plate was derivatized with a 0.05% DPPH methanolic solution and dried for 1 min at room temperature in an extractor hood. The dried plate was maintained in the dark for 30 min. The plate was detected at visible light, and the image was achieved by using the software VisionCats (CAMAG, Muttenz, Switzerland).

### 2.6. Anthocyanins Characterization

#### 2.6.1. Samples Preparation 

The extraction of anthocyanins is commonly performed using acidified methanol or ethanol, to obtain the flavylium cation form, which is stable in acid conditions [30]. Ground whole grain (0.5 g) was extracted with 10 mL of methanol:1 M HCl 85:15 (*v*/*v*) mixture and maintained for 30 min under stirring in the dark. Samples were centrifuged at 8000× *g* for 20 min at 4 °C (Avanti J-25, Beckman Coulter, Brea, CA, USA), passed through a 0.45 μm filter (VWR International, Fontenay-sous-Boys, France), and stored at −20 °C until used. Each extraction was performed in triplicate.

#### 2.6.2. Qualitative LC-MS Analysis

LC/MS analyses were performed by an LCQ fleet (Thermo Fisher, Waltham, MA, USA) spectrometer equipped with a HPLC UltiMate™ 3000 containing a UV–vis detector. The chromatographic column was a Zorbax RX-C18 (2.1 × 150 mm–5 μm) (Agilent, Santa Clara, CA, USA); the linear gradient elution maintained at a flow rate of 0.25 mL min^−1^, was obtained mixing the solutions: (A) water:formic acid 99:1 (*v/v*) and (B) acetonitrile:formic acid 99:1 (*v*/*v*). The gradient was programmed as follows: 0–10 min: 95–80% A, 15–25 min: 80–50% A, 25–32 min: 50% A, isocratic 32–45 min: 50–0% A, and 45–50 min: 0–95% A. The column temperature was 30 °C. The mass spectrometer operated in positive ions in the mass range 50–200 *m*/*z*. 

Chromatograms were recorded fixing the wavelength at 510 nm. Samples were prepared as described in Section 2.4.1 and the anthocyanins identified for comparison with reference standards (cyanidin-3-*O*-glucoside, pelargonidin-3-*O*-glucoside, and peonidin-3-*O*-glucoside) or, when not commercially available, on the basis of data from the literature [31,32,33].

#### 2.6.3. Spectrophotometric Analysis 

The total anthocyanin content of pigmented corn samples was determined according to the AOAC method [34]. The absorbance of samples, prepared as described in Section 2.6.1 and suitably diluted with 0.025M potassium chloride, pH 1, and 0.4M sodium acetate, pH 4.5, buffers, were measured both at 520 and 700 nm, using the last lecture to correct for haze. Each analysis was performed in triplicate. Total anthocyanins (TA) were expressed as cyanidin-3-*O*-glucoside equivalents (CY mg/g), according to the following Equation (1):

TA (CY mg/g) = ΔA × MW × DF × 1000 × V/e × l × W,(1)

where: ΔA is the difference between (A_520nm_–A_700nm_) at pH 1.0 and (A_520nm_–A_700nm_) at pH 4.5; MW is the molecular weight (449.2 g/mol for CY); DF is the dilution factor; 1000 is the factor for conversion from g to mg; V is the extraction volume (L); e is the molar extinction coefficient (26,900 for CY); l is the path length in cm (1 cm); and W is the sample weight (g).

#### 2.6.4. Quantification by HPLC-DAD Method

An HPLC method combined with a diode array detector (DAD) was developed according to the method by Sangiovanni and co-workers, with some modifications [35]. The method was set up according to the Food and Drug Administration (FDA) Guidelines on Bioanalytical Method Validation [36] and validated for anthocyanins (cyanidin, pelargonidin, and peonidin derivatives) quantification by calculating linearity, sensitivity, recovery, and stability of phenolic compounds.

Stock solutions of cyanidin-3-*O*-glucoside (CY), pelargonidin-3-*O*-glucoside (PL), and peonidin-3-*O*-glucoside (PE) were prepared in 0.1 N HCl at a final concentration of 200 μg mL^−1^. The working solutions were prepared in 0.1 N HCl in the concentration range 0.5–10 μg mL^−1^.

The linearity was evaluated considering the correlation coefficient R^2^. Limits of detection (LOD) and quantification (LOQ) were determined at a signal-to-noise ratio of 3 and 10, respectively. Intra-day precision, calculated by the variation coefficient (RSD%) of the peak areas, was determined after five replicates injected in the same day. Inter-day precision was evaluated by repeating the intra-day precision study in three different days. 

The recovery of anthocyanins was evaluated by adding the CY, PL, and PE at three different concentrations (low, medium, and high) to a yellow corn variety. The stability of anthocyanins was assessed in sample extract at −20 °C after different storage times (2, 5, and 20 days).

The anthocyanin separation was performed using an HPLC equipment Jasco (Tokyo, Japan). The instrument consisted of a pump (PU-980, Jasco, Tokyo, Japan), an interface (LC-NETII/ADC, Jasco, Tokyo, Japan), a diode array detector (MD-2010 Plus, Jasco, Tokyo, Japan), a mixer LG-150-0.4 (Jasco, Tokyo, Japan), a degasser (DG-2080-54, Jasco, Tokyo, Japan), and an injection valve (Rheodyne, Cotati, CA, USA) with a 20 μL loop. The chromatographic column was a Synergi 4u MAX-RP 80A (250 × 4.60 mm 4 μm) (Phenomenex, Torrance, CA, USA) with a Security Guard C12 4 × 3.0 mm ID (Phenomenex, Torrance, CA, USA).

The analysis was performed at a flow rate of 0.8 mL min^−1^ using the following gradient: 0–15 min: 94–70%, A, 15–30 min: 70–50% A, 30–35 min: 50–10% A, 35–38 min: 10% A isocratic, and 38–48 min: 10–94% A; where (A) water:acetonitrile:formic acid 96:3:1 (*v*/*v*/*v*); and (B) acetonitrile:water:formic acid 50:49:1 (*v*/*v*/*v*) were used as mobile phases. The detector was set at 520 nm.

### 2.7. In Vitro Gastro-Intestinal Digestion

The stability of anthocyanins at the gastro-intestinal level was tested by simulating the human digestion with the in vitro method previously described, with some modifications [35,37,38].

#### 2.7.1. Samples Preparation 

The gastro-intestinal digestion was applied on the pigmented varieties Sugary, Polenta, and Pop.

Aliquots of 10 g of ground sample were extracted with 100 mL of ethanol:water 50:50 (*v*/*v*) and maintained under stirring for 4 h in the dark. Samples were filtered through a filter paper (Whatman 1 or equivalent). The extractions were repeated for 16 h, and the fractions were combined, evaporated using a Rotavapor, and finally freeze-dried.

#### 2.7.2. Digestion Protocol 

The hydroalcoholic extracts of each sample (100 mg), prepared as described in Section 2.7.1, were added with 6 mL of saliva juice and incubated for 5 min at 37 °C. The gastric digestion was performed by adding 12 mL of gastric juice, and samples were incubated for 2 h at 37 °C. The intestinal digestion was then started by adding 12 mL of duodenal juice and 6 mL of bile juice. Samples were incubated for 2 and 24 h at 37 °C under constant agitation and centrifugated for 5 min at 3000× *g*. The supernatants were freeze-dried, stored at −20 °C until use, and analyzed using the HPLC-DAD method, as described in Section 2.6.4. 

The digestive fluids were prepared as described by Sangiovanni and co-workers [35].

### 2.8. Statistical Analysis

Comparisons of data between pigmented varieties and their related parental varieties were performed by unpaired *t*-test using Welch’s correction. All data are expressed as means with standard deviations (±SD). This statistical test was used to confirm the hypothesis that two populations have equal means. Compared to Student’s *t*-test, it is more reliable when the two samples have unequal variances. When the two-tailed p value was less than 0.05, the difference between the two population of data was considered statistically significant. Statistical analysis was done using GraphPad Prism 8 software (GraphPad Software Inc., San Diego, CA, USA).

## 3. Results and Discussion

### 3.1. Cellular Tests

Although corn (*Zea mays* L.) is one of the most widely used alternatives to gluten-containing grains, this cereal could be responsible for persistent symptoms in very limited subgroups of CD patients [20]. Some authors showed that (1) the digestion of zeins is poor [20,39,40] and (2) after deamidation by trasglutaminase-2 (TG2), corn prolamins can be recognized by IgA contained in the sera of some CD patients [41]. In a study performed by Bergamo and co-workers, intestinal T-cells from seven CD subjects were treated in vitro with corn prolamins. One patient produced IFN-γ as a result of T-cell stimulation. Although this response was not specific, corn and teff prolamins produced higher levels of IFN-γ (145.6 and 154.4 pg/mL, respectively) compared to the medium (10.9 pg/mL) and other “non-toxic” grains such as millet and quinoa (≈110 pg/mL) [42]. Although corn is generally safe for CD subjects, some patients do not respond to the gluten-free diet. This could be attributable to a higher sensibility of these patients to gluten contamination or to the presence of other cereal prolamins, such as zeins [20].

On these bases, it is important to evaluate the safety of new varieties, such as the pigmented ones.

In the myelogenic leukemia cells K562(S), the toxic gliadin peptides are able to activate the TG2, an enzyme involved in the precocious events associated with CD and that leads to a dramatic increase in intracellular ROS [16,23,43]. In detail, the p31–43 fragment is involved in the Ca^2+^-dependent TG2 activation, determining the cytoskeleton rearrangement and, as a consequence, the cell agglutination. Therefore, this cell line represents a useful and rapid tool for evaluating the activation of epithelial cells in CD, mediated by cereals.

The K562(S) cells were incubated with cereal samples after in vitro proteolysis, and the percentage of agglutinated cells were determined. The higher the percentage of agglutination, the higher is the ability of samples to induce the TG2 activation. The rice flour, used as a negative control, did not induce K562(S) cell agglutination, whereas the positive control (wheat flour) determined a massive agglutination. The exposure of K562(S) cells to the product of digestion from both pigmented corn (Reduno and Polenta) did not produce cell agglutination (Table 1).

As for the K562(S) cells, the T84 cell line allows the evaluation of the gliadin-dependent early events [44]. When T84 cells are grown on a polycarbonate filter in a bidimensional cell culture system, the fragment p31–43 causes the cytoskeleton rearrangement determining an increased permeability of the cell mono-layer [23]. Table 1 shows the results on the TEER of a T84 cell monolayer after a 3 h incubation with the cereal samples coming from the proteolysis.

The incubation of the cell culture system with the wheat sample determined a significant decrease in TEER with a consequent increase in TEERsample/TEERcontrol. The corn samples did not affect the TEER values, which were comparable to the negative control (rice).

All the events investigated in this in vitro study were TG2-dependent. The pigmented corn tested did not impact the TG2-mediated cytoskeleton rearrangement in K562(S) and T84 cell lines, confirming their safety for celiac patients. The inclusion of these pigmented varieties in the gluten-free diet could be considered safe and suitable to improve celiac subjects’ health, thanks to their nutritional and functional properties. To better characterize the pigmented corn varieties, samples included in this study were chemically characterized and studied for their antioxidant activity, applying both spectrophotometric and chromatographic techniques.

### 3.2. Polyphenols Content

**Figure 2:**
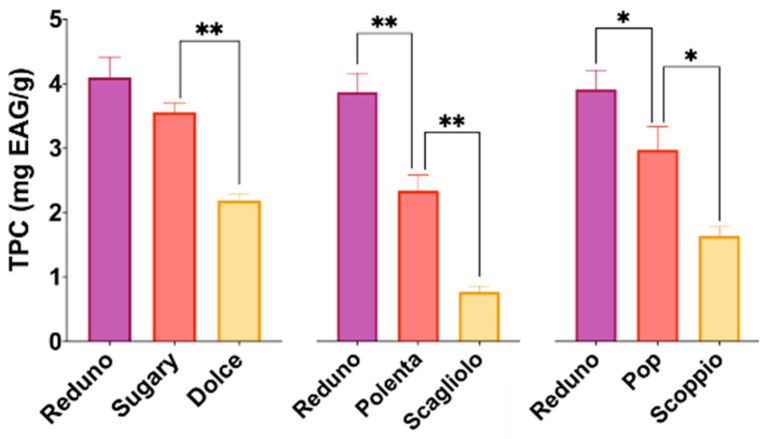
Soluble phenolic content (SPC) expressed as mg GAE/g (* *p* < 0.05, ** *p* < 0.01).

Cereals contain phenolic compounds in both soluble and bound forms; the latter are associated with the cell wall components. Some studies suggest that the soluble fraction is an excellent source of dietary antioxidants, [45] and, for this reason, the study here reported focused attention on it. Among cereals, the pigmented corn varieties, rich in soluble phenolic compounds, could be considered as interesting gluten-free ingredients. To evaluate the beneficial and functional properties of pigmented corn for celiac patients, seven samples (among them were some yellow varieties for comparison) were included in this research. The soluble phenolic content (SPC) of samples, determined by the Folin–Ciocalteau’s assay, ranged between 0.81 ± 0.10 and 4.10 ± 0.32 mg GAE/g of grain flour (Figure 2).

The soluble phenolic contents of pigmented varieties (Reduno, Sugary, Polenta, and Pop) were higher than those measured in yellow ones, probably due to the contribution of anthocyanins. These values are in agreement with data by Lopez-Martinez and co-workers [24], where the soluble polyphenol content in corn samples (with different pigmented phenotypes) ranged between 0.33 and 6.80 mg GAE/g and those by Suriano and co-workers (2021), where the soluble polyphenol content in one yellow and three pigmented corn varieties ranged between 1.36 and 4.05 mg catechin equivalents/g [46]. Lower values were found by Rodríguez-Salinas and co-workers (2019), who reported a soluble polyphenol content in corn samples (15 samples from white to purple varieties) between 1.05 and 1.39 mg GAE/g [47].

The samples Reduno and Sugary showed the highest SPC (4.1 ± 0.32 and 3.56 ± 0.13 mg GAE/g, respectively). All the pigmented varieties showed an improvement, in terms of soluble phenolic compounds, compared to the corresponding parental yellow ones (*p* < 0.05 for Pop and *p* < 0.01 for Sugary and Polenta).

The Folin–Ciocalteau’s method is a rapid and useful assay for collecting preliminary data on the phenolic content of a sample, although some molecules (e.g., ascorbic acid and aromatic amines) could interfere with this method.

### 3.3. Antioxidant Capacity

The identification of new gluten-free ingredients containing healthy compounds could contribute to the diversification and positive properties of the gluten-free diet. In this contest, it is important to evaluate the antioxidant capacity associated with these new ingredients.

**Figure 3:**
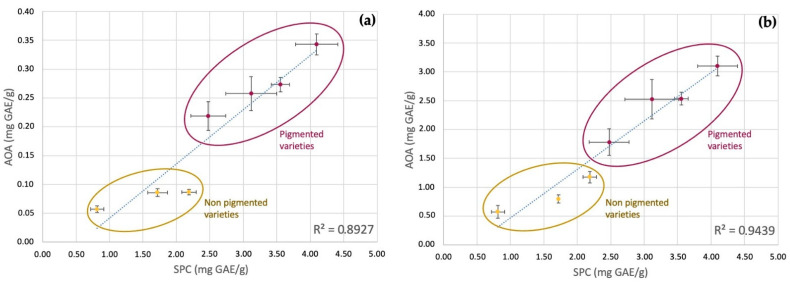
Correlation between soluble phenolic content (SPC) and the antioxidant activity (AOA) measured with: (**a**) DPPH and (**b**) TEAC assays.

Data on antioxidant activity for each sample, measured with DPPH and TEAC assays, were correlated to the soluble polyphenol content, as in Figure 3. The antioxidant capacity values ranged between 0.06–0.34 mg GAE/g and 0.57–3.10 mg TE/g, respectively. 

Generally speaking, the phenotypes with the highest DPPH scavenging activity were those of higher ABTS-reducing activity. The differences in the antioxidant capacity among non-pigmented and pigmented varieties could be mainly due to the presence of anthocyanins with known antioxidant capacity [18]. 

Samples with the highest content of phenolic compounds also showed the highest antioxidant capacity: in fact, a good correlation between the soluble phenolic content (SPC) and the antioxidant activity (AOA), measured with DPPH and ABTS tests, was observed (R^2^ > 0.89).

Phenolic profile and antioxidant activity were also correlated by HPTLC. To obtain a semi-quantitative evaluation of the antioxidant capacity of samples, the DPPH was selected as a derivatization agent, and the plate was detected at visible light.

**Figure 4:**
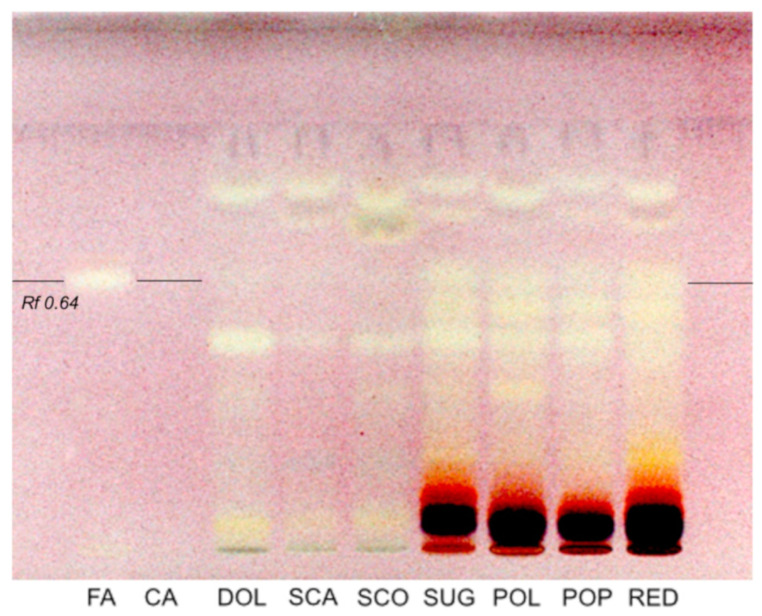
HPTLC patterns of soluble phenolic fractions at visible light after derivatization with DPPH solution. Standard phenolic acids were run in parallel (FA: ferulic acid, CA: coumaric acid, DOL: Dolce, SCA: Scagliolo, SCO: Scoppio, SUG: Sugary, POL: Polenta, POP: Pop, RED: Reduno).

The variation of color, from violet to yellow, was proportional to the AOA of each phenolic compound (Figure 4).

Coumaric acid did not show antioxidant activity, while the complex fingerprints of samples decreased the possibility to identify all bands separated during the chromatographic run. Bands with the same *Ratio frontis* (Rf) of ferulic acid (Rf = 0.64) were well visible in all pigmented samples (Sugary, Polenta, Pop, and Reduno). Pigmented corns, in particular, the purple varieties, generally showed the higher content of phenolic compounds [46], which is mainly correlated to the presence of anthocyanins [48,49]. Although this method does not take into account the contribution of anthocyanins to the AOA (due to the dark color of these molecules), it is interesting to note that the purple corn Reduno and the pigmented samples showed the highest content of antioxidant compounds compared to the yellow ones. 

### 3.4. Anthocyanin Characterization

Pigmented corn varieties are characterized by a complex anthocyanin profile. Anthocyanins provide different coloring to the grain, from red to purple. These compounds exert well-known antioxidant properties, and they are able to scavenge free radicals and protect cells against membrane damage [18].

To investigate the role of anthocyanins in the modulation of chronic inflammation in celiac subjects, it is important to characterize their profile in corn samples.

Due to their similar spectral characteristics and the limited commercial availability of purified compounds, HPLC coupled to mass spectrometry was used for the characterization of anthocyanins profile in samples. The identification of each anthocyanin was based on the comparison with commercial standards and, when not available, on data from the literature. According to the literature, the main anthocyanins in corn are the glucoside and malonyl derivatives of cyanidin, pelargonidin, and peonidin [31,32,33,50,51,52,53,54]. The compounds identified by LC-MS are reported in Table 2. Anthocyanin profiles are very similar among pigmented samples. Differences were observed only in the Polenta variety, where pelargonidin-3-*O*-(6′′-malonyl-glucoside) and peonidine-3-*O*-(6′′-malonyl-glucoside) were not detectable.

Table 3 lists the validation parameters for the HPLC-DAD method developed in this study to quantify the main anthocyanins present in corn (cyanidin-3-*O*-glucoside, pelargonidin-3-*O*-glucoside, peonidin-3-*O*-glucoside). 

The precision of a chromatographic method is the agreement between a series of measurements of the same sample obtained under the prescribed conditions. The coefficient of variation (CV%) between the measurements must be within the ±15% [36]. As reported in Table 3, the method is precise (CV% < 15%). The sensitivity of method, which indicates the lowest concentration of analyte, which can be detected (LOD) or quantified (LOQ), was tested, and the data are listed in Table 3.

**Figure 5:**
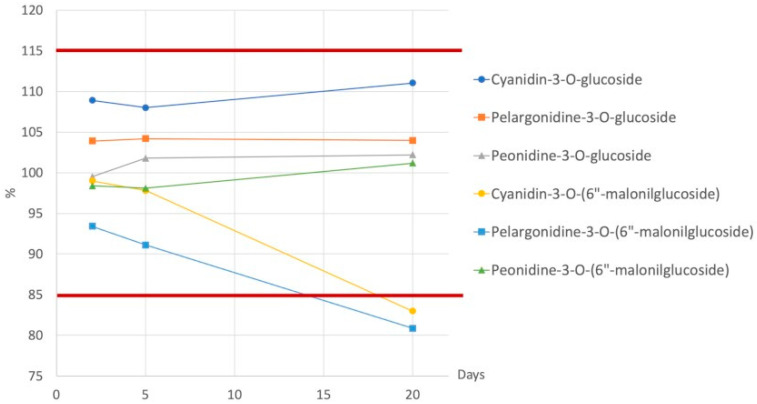
Percentage of variation of anthocyanins evaluated maintaining samples at −20° for 2, 5, and 20 days.

The recovery, that is the efficiency and reproducibility of extraction, needs to be consistent and reproducible [36]. The recovery ranged between 80 and 115% and complied with FDA recommendations [36]. The stability of compounds was evaluated maintaining the extracted samples at −20 °C for different times (2, 5, and 20 days), and the results are shown in Figure 5. The glucoside derivatives were more stable compared to the malonyl derivatives; to guarantee a percentage of variation within ±15%, samples had to be stored at −20° and analyzed within 5 days from the extraction.

**Figure 6:**
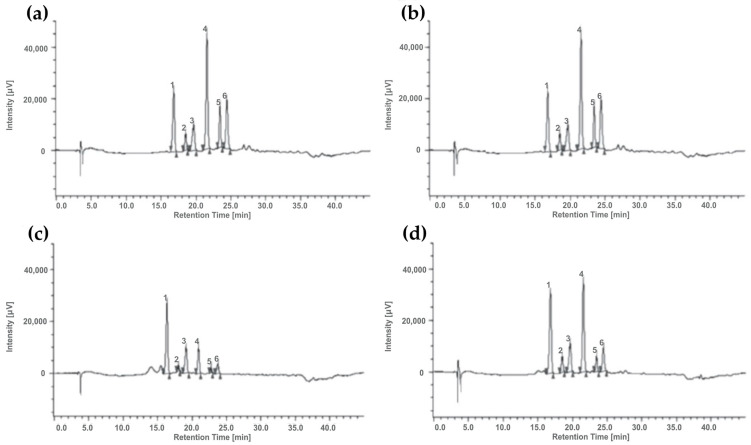
Chromatographic profiles of anthocyanins in (**a**) Reduno, (**b**) Pop, (**c**) Polenta, and (**d**) Sugary samples: (1) cyanidin-3-*O*-glucoside; (2) pelargonidin-3-*O*-glucoside; (3) peonidin-3-*O*-glucoside; (4) cyanidin-3-*O*-(6′′-malonil-glucoside); (5) pelargonidin-3-*O*-(6′′-malonil-glucoside); (6) peonidin-3-*O*-(6′′-malonil-glucoside).

Figure 6 shows some examples of chromatograms obtained by the HPLC-DAD. 

Table 4 compares the total anthocyanin content, quantified by spectrophotometric method and HPLC-DAD chromatography. Cyanidin-3-*O*-(6′′-malonyl-glucoside), pelargonidin-3-*O*-(6′′-malonil-glucoside), and peonidin-3-*O*-(6′′-malonil-glucoside) were not commercially available so that these molecules were quantified as equivalents of the corresponding glucoside derivative.

When measured spectrophotometrically, the total anthocyanin content (TA) of purple corn Reduno was significantly higher than those of pigmented varieties: Polenta (*p* < 0.0005), Pop (*p* < 0.005), and Sugary (*p* < 0.0001). This is in agreement with the total anthocyanin content of 0.015–8.60 mg CY/g, previously reported by Lopez-Martinez and co-workers [24], and the total anthocyanin content of 0.014–0.74 mg CY/g shown by Rodríguez-Salinas and co-workers [47].

Similar results were obtained with the HPLC-DAD technique. A good correlation between values from the two analytical methods was obtained being R^2^ > 0.98. The total anthocyanin concentration ranged between 286.0 ± 22.3 and 568.8 ± 46.1 μg/g, in agreement with the study by Suriano and co-workers (2021), where anthocyanin content in blue and purple corn varieties ranged between 167.5 and 711 μg/g [46]. In all samples, cyanidin-3-*O*-glucoside was the most abundant compound apart from Pop, which was richer in cyanidin-3-*O*-(6′′-malonyl-glucoside) (132.4 ± 11.7 μg/g). Cyanidin derivatives ranged between 49 and 65% of total anthocyanins, data slightly lower than those reported by other authors, where the cyanidin derivatives represented about 70% of total anthocyanins [50].

Peonidin derivatives constituted between 30–44% of total anthocyanins in Sugary and Reduno, respectively. As expected, the parental purple corn Reduno showed the highest anthocyanin content.

Corn is one of the ingredients most frequently used for the formulation of gluten-free products. A study performed in 1992 and based on 6218 people (age: 1–92 years) reported a consumption of corn by the general population of 3 g/day in the Netherlands [55,56] and about 7.2 g/day in the EU [56,57]. The consumption of corn was also calculated for subjects suffering from gluten-related disorders (celiac disease or Dühring’s disease). Since, in this group of people wheat, barley and rye were replaced by gluten-free cereals, the consumption of corn was 162 g/person/day [55,56].

As reported in the scientific literature, the level of anthocyanins in pigmented corns (from pink to purple) ranges between 0.09 and 0.96 mg/g [4]; our experimental data on Polenta, Pop, and Sugary varieties are in agreement since the total anthocyanin content was 0.48–0.69 CY mg/g. Considering the consumption of corn by celiac patients, the daily intake of anthocyanins associated with the pigmented varieties reported above could range between 77.8 and 111.8 mg/days. 

Different in vivo and in vitro studies showed a role of anthocyanins in modulating inflammatory process and oxidative stress [58]. The reduction in some inflammatory markers was observed with polyphenol concentrations comparable to those measured in the cereals included in this study. The administration of 10 g of strawberry powder (containing 94.7 mg of total polyphenols, including anthocyanins) determined after 6 h a significant reduction in the plasma interleukine-6 (IL-6) and high-sensitivity C-reactive protein (hsCRP) [59]. The same extract decreased postprandial IL-1β and plasminogen activator inhibitor-1 (PAI-1) after six weeks of consumption [60]. 

In another intervention study, increasing doses of a cranberry juice for four weeks (from 125–500 mL/day, containing from 100 to 400 mg of total phenols) showed, when compared to the baseline, a significant reduction in plasma-soluble vascular adhesion molecule (sVCAM-1), soluble intercellular adhesion molecule (SICAM-1), and matrix metalloproteinase (MMP-9) [61,62].

### 3.5. In Vitro Gastro-Intestinal Digestion

**Figure 7:**
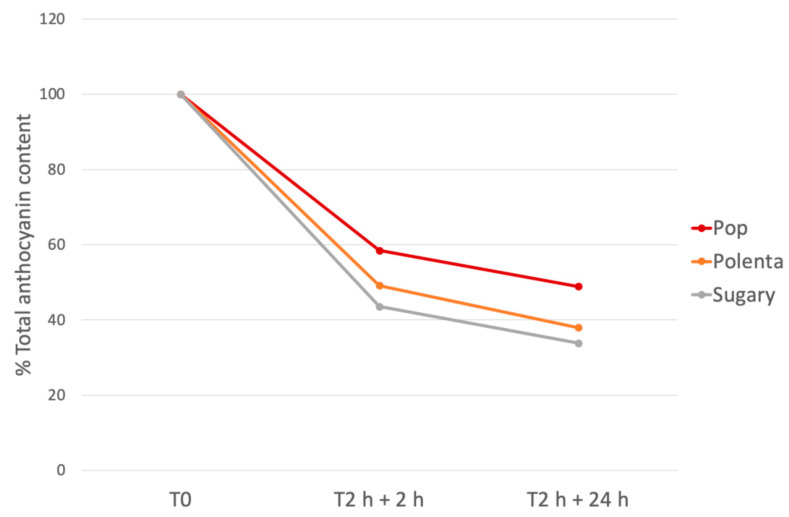
Reduction trend (in percentage) of total anthocyanin content during the gastro-intestinal digestion. POP: Pop; POL: Polenta; SUG: Sugary. T0: before digestion; T2 h + 2 h: 2 h of gastric digestion + 2 h of intestinal digestion; T2 h + 24 h: 2 h of gastric digestion + 24 h of intestinal digestion.

In order to evaluate the fate of anthocyanins during the gastro-intestinal digestion, an in vitro protocol was applied to corn varieties suitable for human consumption. The reduction in anthocyanins (as a percentage of starting concentration) during the in vitro gastro-intestinal digestion is illustrated in Figure 7.

Gastrointestinal digestion determined a significative reduction in anthocyanins. After 2 h of gastric and 2 h of intestinal digestion, the percentage of reduction was 38.1 ± 1.8% for pelargonidin-3-*O*-(6′′-malonyl-glucoside) in the Pop sample and 59.8 ± 0.8% for pelargonidin-3-*O*-glucoside in the Polenta sample. After 2 h + 24 h of gastrointestinal digestion, the percentage of reduction was 45.2 ± 7.6% for pelargonidin-3-*O*-(6′′-malonil-glucoside) in the Pop sample and 83.0 ± 1.2 for peonidin-3-*O*-(6′′-malonyl-glucoside) in the Sugary pigmented variety. It is interesting to note that the anthocyanin degradation occurred mainly within the first two hours of intestinal digestion (2 h + 2 h) (Figure 7).

The anthocyanin stability could be influenced by different factors such as the pH, the presence of oxygen, their chemical structure, and the action of different enzymes [63]. The stability of anthocyanins under the gastric conditions was confirmed by several studies [64,65]: the pH of the stomach (1–2) maintains anthocyanins in their flavylium cation, which is the most stable form. On the contrary, the pH of the intestine, close to neutrality, and the activity of human microbiota convert anthocyanins into several metabolites, such as their corresponding phenolic acids and aldehydes [66,67].

The potential health benefits of anthocyanins from cereals was the object of extensive research; however, many studies on pigmented cereals were performed with extracts or pure anthocyanin compounds [21]. Food processing and digestion could modulate the beneficial effect of these compounds, and, in particular, the stability of anthocyanins during the gastro-intestinal digestion is important to guarantee their bioavailability and, as a consequence, the possible protective role at the gastro-intestinal level. The stability of corn anthocyanins during the digestion process, observed in this research, correlated well with data obtained in other food matrices. Sangiovanni and co-workers [35] studied the biological activity of a *Vitis vinifera* L. leaves extract. The gastrointestinal digestion of this extract determined an anthocyanin reduction ranging between 38 and 71% for cyanidin-3-*O*-glucoside and delphinidin-3-*O*-glucoside, respectively. David and co-workers [68] observed a reduction of 70% of total anthocyanins, after the gastro-intestinal digestion of a *Cornus mas* L. extract.

## 4. Conclusions

The number of people with intestinal and extra-intestinal symptoms associated with the consumption of gluten has increased in the last decades; amongst GRDs, the CD is the most frequent clinical form. A strict and life-long GFD is the only currently available treatment for CD. However, it is associated with many issues: firstly, gluten-free products lack both palatability and fragrance; in addition, GFD could be associated with nutrient imbalance in the long term [2]. All these findings underline the need for an improvement in the nutritional and sensorial quality of gluten-free products, taking also into consideration that the mucosal inflammation in CD is likely to persist in most celiac individuals on a GFD [69]. In recent years, the interest in the health benefits of dietary antioxidants has been increased contributing to the rapid diffusion of functional foods. Thanks to their richness in antioxidants, the pigmented corn varieties could be considered an interesting “new” ingredient for gluten-free products, although the technological performance of their flour must be confirmed. Since the oxidative stress at the intestinal level can persist in celiac subjects who maintain GFD, pigmented corn varieties could counteract this phenomenon and for this reason were included in this study. The samples included in this study were characterized in terms of phenolic compound content and antioxidant capacity. As expected, the pigmented varieties, rich in anthocyanins, showed a significantly higher content of polyphenols than those of the common yellow varieties and, as a consequence, a potential higher antioxidant/protective activity. Although the in vitro digestion determined a reduction in the content of anthocyanins, it is interesting to note that the anthocyanin degradation occurred mainly within the first two hours of intestinal digestion so that the activity in this area could be preserved for a certain period of time. Other investigations on the in vivo bioavailability of anthocyanins and their stability to technological treatments in food preparation (like cooking processes) are necessary to study their influence in health properties and are in progress in our laboratory.

The cellular tests applied in this study confirmed the lack of alteration by the pigmented varieties in cell monolayer permeability, which is a key step in the mucosal inflammatory cascade in CD. On these bases, the safety of the tested cultivars for celiac patients seems confirmed.

Considering the daily consumption of corn and the high content of phenolic compounds and anthocyanins in pigmented corn, these varieties could contribute to supply antioxidant and anti-inflammatory ingredients to the diet of the general population but in particular of the celiac subjects, since corn represents one of the most important ingredients among cereals used in the formulation of gluten-free products. 

## Figures and Tables

**Table 1:** Measurement of % of agglutinated K562(S) cells and transepithelial electrical resistance (TEER, ΔΩ ms/mm^2^) incubated with the digested products from cereals.

Digested Sample	% of Agglutination			TEER (TEER_sample_/TEER_control_)	
	1	2	3	1	2
Wheat	90	78	94	3.3	4
Rice	0	0	0	1	-
Reduno	0	0	0	1	0.9
Polenta	0	0	0	1	1

**Table 2:** Anthocyanins identified in pigmented samples.

	*m*/*z*	Samples			
		RED	SUG	POL	POP
Cyanidin-3-*O*-glucoside	449	+	+	+	+
Pelargonidin-3-*O*-glucoside	493	+	+	+	+
Peonidin-3-*O*-glucoside	463	+	+	+	+
Cyanidin-3-*O*-(6′′-malonil-glucoside) *	535	+	+	+	+
Pelargonidin-3-*O*-(6′′-malonil-glucoside) *	519	+	+	-	+
Peonidin-3-*O*-(6′′-malonil-glucoside) *	549	+	+	-	+

* Compounds identified on the basis of literature data; RED: Reduno, POL: Polenta, POP: Pop, SUG: Sugary. +: Detectable; -: Undetectable.

**Table 3:** HPLC-DAD validation parameters for the analysis of anthocyanins.

	Precision ^a^		Linearity		Sensitivity				Recovery ^#^
	Intraday (CV%)	Interday (CV%)	Linear Range (μg/mL)	R^2^	LOD		LOQ		% (mean ± SD)
					(μg/mL)	(μg/g)	(μg/mL)	(μg/g)	
CY	2.44	5.79	0.5–10	0.9987	0.04	0.86	0.15	3.00	92.2 ± 3.6
PL	2.06	4.77	0.5–10	0.9951	0.07	1.49	0.25	5.00	88.4 ± 7.0
PE	3.25	4.17	0.5–10	0.9972	0.14	2.79	0.50	10.00	96.3 ± 6.4

CY: cyanidin-3-*O*-glucoside, PL: pelargonidin-3-*O*-glucoside, PE: peonidin-3-*O*-glucoside. ^a^ n = 5; ^#^ n = 3.

**Table 4:** Total anthocyanins (TA) measured by the AOAC spectrophotometric method and HPLC-DAD technique (n = 3).

	TA (CY mg/g) (mean ± SD)	HPLC-DAD Analysis (μg/g) (mean ± SD)						
		CY	PL	PE	CYm	PLm	PEm	Total
RED	0.98 ± 0.03	176.0 ± 11.4	24.5 ± 1.9	155.8 ± 14.8	100.4 ± 8.2	14.7 ± 1.4	97.4 ± 10.3	568.8 ± 46.1
POL	0.48 ± 005	142.6 ± 11.0	9.6 ± 1.2	74.2 ± 4.9	37.8 ± 3.5	NQ	21.8 ± 2.4	286.0 ± 22.3
POP	0.69 ± 0.05	75.9 ± 5.4	16.4 ± 0.1	56.7 ± 3.4	132.4 ± 11.7	39.5 ± 3.7	90.3 ± 6.3	411.1 ± 29.8
SUG	0.57 ± 0.03	114.7 ± 5.3	17.3 ± 2.1	64.5 ± 2.8	112.7 ± 3.9	13.7 ± 0.8	46.6 ± 1.9	369.5 ± 15.7

CY: cyanidin-3-*O*-glucoside, PL: pelargonidin-3-*O*-glucoside, PE: peonidin-3-*O*-glucoside, CYm: cyanidin-3-O-(6′′-malonyl-glucoside), PLm: pelargonidin-3-*O*-(6′′-malonyl-glucoside), PEm: peonidin-3-*O*-(6′′-malonyl-glucoside), NQ: not quantifiable <LOQ (5 μg/g). RED: Reduno, POL: Polenta, POP: Pop, SUG: Sugary.

## Data Availability

The data presented in this study are available within this article.
